# Are we ready for routine precision medicine? Highlights from the Milan Summit on Precision Medicine, Milan, Italy, 8–9 February 2018

**DOI:** 10.3332/ecancer.2018.817

**Published:** 2018-03-05

**Authors:** Luca Mazzarella

**Affiliations:** European Institute of Oncology, Via Ripamonti 435, Milan 20141, Italy

**Keywords:** genomics, next generation sequencing, NGS, precision medicine, targeted therapy

## Abstract

On 8 and 9 February 2018, the IFOM-IEO campus in Milan hosted the Milan summit on Precision Medicine, which gathered clinical and translational research experts from academia, industry and regulatory bodies to discuss the state of the art of precision medicine in Europe. The meeting was pervaded by a generalised feeling of excitement for a field that is perceived to be technologically mature for the transition into clinical routine but still hampered by numerous obstacles of a methodological, ethical, regulatory and possibly cultural nature. Through lively discussions, the attendees tried to identify realistic ways to implement a technology-rich precision approach to cancer patients.

## Introduction

The concept of ‘precision medicine’, as highlighted by Professor De Braud (Istituto Nazionale dei Tumori (INT), Italy) at the summit, has transitioned in recent years from an initial focus on tumour genetic profiling and targeted drugs to a broader definition encompassing multiple phases of cancer natural history, including prevention, early diagnosis, therapy and surveillance. Although several studies have shown that a precision medicine approach can significantly improve patient outcomes, widespread adoption is made difficult by the complex, multidisciplinary nature of the required expertise and the need for adequate support of a financial, organisational and administrative nature. The summit brought together experts with heterogeneous backgrounds to discuss how to overcome current barriers. Many speakers provided an overview of ongoing large-scale multi-institutional projects assessing the feasibility and clinical benefit of next-generation sequencing (NGS) and other high-throughput molecular analytical approaches. An overview of recent technological improvement was provided, especially on the growing sophistication and utility of liquid biopsies. Finally, some speakers concentrated on novel biological understanding obtained from molecular characterisation.

## Multi-institutional precision medicine projects

Large-scale sequencing projects are being conducted in several countries [1]. Professor PG Pelicci (European Institute of Oncology (IEO), Italy) reviewed the experience of the Alleanza Contro il Cancro (ACC) consortium in Italy, which groups together 21 cancer centres. ACC recently launched a nationwide pilot trial in lung cancer that aims at collecting and analysing detailed genetic information from 1,000 patients, using a custom-designed targeted sequencing panel focused on actionable mutations. Beside evaluating the feasibility and accuracy of large-scale sequencing in a disease in which genetic stratification is a key to adequate treatment, the programme will systematically collect samples for longitudinal studies on biomarkers of treatment response. The consortium will then apply an evolution of this approach to multiple other diseases, collecting all clinical and medical information in a national repository that will allow the development of a computational platform to assist in clinical decision making and will accelerate patient enrolment to clinical trials. Similar efforts are ongoing in Spain, as highlighted by Dr Garrido (Universidad de Alcalá, Madrid, Spain) [2].

Dr Tonu Esko (Estonian Genome Center, Estonia) provided an overview of the Estonian program for personalised medicine, which aims to obtain detailed molecular profiling of 52,000 adults (5% of the adult population) followed longitudinally with direct matching to electronic medical records. Thousands of these subjects are expected to be characterised by whole genome/whole exome sequencing, transcriptomics and metabolomics. Although the ethical implications of matching genetic information with medical information are evident, a survey showed that an overwhelming majority (>70%) of interviewees support the project, suggesting that ethical concerns can be overcome if the expected return is tangible.

Dr Kramer highlighted the role that molecular profiling based on gene expression [3, 4] or DNA methylation [5] can have in identifying the tissue of origin and best treatment in cases of cancer of unknown primary, a heterogeneous group of diseases for which the current median overall survival is in the order of a few months [6]. NGS of tumour DNA [7] and analysis of cfDNA [8] can identify actionable mutations and elevated tumour mutational burden [9] in ~10–30% of the cases, offering a valid therapeutic alternative to chemotherapy as demonstrated by several case reports [10, 11]. Strategies to personalise treatment based on gene expression [9] or NGS (MX39795 trial) are ongoing.

Dr Andrew Hughes from the Christie Hospital (Manchester, UK) provided an overview of the TARGET trial in the UK, aimed at validating liquid biopsy for metastatic disease monitoring and treatment allocation. He highlighted six groups of issues faced by precision medicine programmes: ethical, administrative, technological, scientific, financial and organisational. Besides scientific and technological issues related to reliability of the assay and interpretation of the result, discussed by many others, he identified other crucial aspects worth mentioning. First, the crucial ethical issue of managing information returns to the patient, which TARGET decided to deliver using the ‘flexible-default’ model [12, 13]. Second, the availability of electronic databases and medical records, which are key to allowing information sharing and decision making within the Tumour Molecular Board. Finally, the importance of activating a fair number of biomarker-driven clinical trials to allocate patients based on the molecular profile: increase in trial availability resulted in an increased allocation rate from 0% in 2012 to 18% in 2016. This translates into a real clinical benefit for patients, as meta-analyses of phase 1, 2 and 3 trials demonstrate an improvement in outcome measures when patients are allocated to biomarker-driven trials, as opposed to nonbiomarker-driven [14–16]. A sobering point of view was provided by Dr Le Tourneau (Institut Curie Paris, France) who pointed out that much of the benefit observed in these meta-analyses is actually limited to FDA-approved drugs and is less clear for experimental drugs at earlier phases of development. Still, many attendees expressed the view that current access to targeted therapy is inadequate and, in light of the demonstrated clinical benefit, increased reimbursement for high-throughput molecular testing is justified.

Talks by Professor De Braud, Dr Biganzoli (University of Milan, Italy) and Professor Curigliano (European Institute of Oncology (IEO), Italy) dwelled on the changing face of drug development in the framework of personalised medicine, with an increased usage of adaptive trial designs that allow testing of multiple hypotheses at once, so as to maximise costs and patient enrolment [17]. The speakers called for a new comprehensive strategy that overcomes the current single drug-driven model, a strategy best promoted by academia, regulatory bodies and the government in collaboration with pharmaceutical companies.

Two talks were held by representatives of the European (EMA) and Italian (AIFA) regulatory bodies. A hotly discussed topic was that of histology-agnostic biomarkers. Even if considered very biologically relevant, many are insufficiently supported by clinical trials to be clearly recommended into clinical practice. Prominent examples are programmed death ligand 1 (PDL1) expression, troubled by inter-assay technical variability and a heterogeneous predictive power in different diseases [18] and BRCA1/2 mutational status, insufficient to identify responders to poly-ADP ribose polymerase inhibitors [19].

An intriguing talk by the psychologist Ketti Mazzocco (University of Milan, Italy) highlighted the importance of ‘framing’ information to patients and how appropriate communication strategies can increase the chances of a patient’s acceptance of precision medicine approaches.

## Technological advancements

Drs Ross and Pavlick from Foundation Medicine, the private company that pioneered implementation of NGS for precision oncology, shared their experience on thousands of patients analysed through their commercially available platforms. Their NGS-based assay led to the identification of several genetic alterations actionable for targeted treatment [20–22]. They identified many technical barriers to precision medicine: the inter- and intra-individual genomic complexity, requiring adequately sized sequencing panels; the difficulty in obtaining adequate bioptic material; assay sensitivity, crucial since false negatives will cause patients to be treated with significantly more toxic and/or less effective therapy. Discordance rate between NGS-based and conventional, approved non-NGS analytical techniques is extremely high, up to 35% for anaplastic lymphoma kinase translocations.

A wide overview on liquid biopsy was presented by Dr Klaus Pantel (University Medical Centre Hamburg-Eppendorf, Germany), a pioneer in the field. Liquid biopsy is an umbrella name that encompasses different assays detecting circulating tumour cells (CTC), tumour DNA (ctDNA) or other biologically relevant moleculaes (microRNAs, exosomes, metabolites, etc.). Liquid biopsies can provide useful information in various oncological settings: early detection and screening, prognostication by quantifying risk of relapse/progression, real-time monitoring of therapies and identification of resistance mechanisms [23, 24]. Perhaps a poorly recognised issue for early detection/screening is the high rate of background positivity for some common mutations such as TP53 [25, 26], probably reflecting precancerous lesions in apparently healthy subjects. A combination of multiple markers is likely to provide increased specificity [27]. In the setting of tumour prognostication, CTCs are certainly the most developed technology [28, 29], to the point that some authors advocate implementing CTC counts in the TNM staging system. Additional useful biological information can be retrieved from CTCs, to be used to drive therapy with immune checkpoint inhibitors (like PDL1 expression) or to better decide on endocrine therapy in metastatic breast cancer, since CTC often show heterogeneous estrogen receptor status [30, 31]. CtDNA analysis has become established in specific settings, like the identification of T790M resistance-inducing mutations in epidermal growth factor receptor (EGFR)-mutated lung cancer. However, the currently approved methodology remains poorly sensitive, with up to 30% false-negatives [32]. But as pointed out by Jeff Ross from Foundation Medicine, the information retrievable from cfDNA-based liquid biopsies goes beyond individual mutational hotspots, including for instance Tumour Mutational Burden that can identify responders to immune checkpoint inhibitors [33].

## Biological advancements

Professor Foiani from the IFOM institute in Milan discussed newly discovered roles of ataxia telangiectasia and Rad3-related protein/ataxia telangiectasia mutated (ATR/ATM) kinases, well known to be involved in maintenance of DNA stability and recently found by himself and colleagues to play a role in tumour migration and metastasis [34].

Dr Bedognetti (Sidra Medicine, Doha, Qatar) reviewed the progress brought about by NGS in the understanding of tumour microenvironment and immunity. RNA expression studies identified gene modules over-represented in various immune phenomena (like autoimmunity or rejection) and associated with different outcomes and/or response to immunotherapy in multiple tumours, which are referred to as immunological constant of rejection (ICR). By analysing these modules in breast cancer, they identified 4 ICR subgroups (ICR1-4) that variably correlated with genomic features associated with differential response to immunotherapy (mutational burden, specific mutations in Molecular Analysis for Personalized Medicine kinases, structural aberrations) [35]. These gene expression-based profiles are evolving into clinical-grade assays to be used for predicting sensitivity to immunotherapy [36], especially in poorly immunogenic diseases like breast cancer. This and similar studies have led to novel therapeutic combinations that may turn immunologically ‘cold’, nonreponsive tumours into ‘hot’, immune therapy-sensitive ones [37]

## Funding

The author was remunerated for writing this conference report through an unrestricted educational grant provided by Roche Foundation Medicine.
